# Impact of data model and point density on aboveground forest biomass estimation from airborne LiDAR

**DOI:** 10.1186/s13021-017-0073-1

**Published:** 2017-02-15

**Authors:** Mariano Garcia, Sassan Saatchi, Antonio Ferraz, Carlos Alberto Silva, Susan Ustin, Alexander Koltunov, Heiko Balzter

**Affiliations:** 1 Jet Propulsion Laboratory (JPL), California Institute of Technology, Pasadena, CA 91109 USA; 2 Department of Geography, Centre for Landscape and Climate Research, University of Leicester, Leicester, LE1 7RH UK; 3 US Forest Service (USDA), Rocky Mountain Research Station, RMRS, 1221 South Main Street, Moscow, ID 83843 USA; 4 Department of Forest, Rangeland, and Fire Sciences, College of Natural Resources, University of Idaho, (UI), 875 Perimeter Drive, Moscow, ID 83843 USA; 5 Center for Spatial Technologies and Remote Sensing (CSTARS), University of California Davis, Davis, USA; 6 National Centre for Earth Observation, University of Leicester, Leicester, LE1 7RH UK

**Keywords:** Airborne LiDAR data, Aboveground biomass, Point density, Data thinning, Echo-based, Canopy height model

## Abstract

**Background:**

Accurate estimation of aboveground forest biomass (AGB) and its dynamics is of paramount importance in understanding the role of forest in the carbon cycle and the effective implementation of climate change mitigation policies. LiDAR is currently the most accurate technology for AGB estimation. LiDAR metrics can be derived from the 3D point cloud (echo-based) or from the canopy height model (CHM). Different sensors and survey configurations can affect the metrics derived from the LiDAR data. We evaluate the ability of the metrics derived from the echo-based and CHM data models to estimate AGB in three different biomes, as well as the impact of point density on the metrics derived from them.

**Results:**

Our results show that differences among metrics derived at different point densities were significantly different from zero, with a larger impact on CHM-based than echo-based metrics, particularly when the point density was reduced to 1 point m^−2^. Both data models-echo-based and CHM-performed similarly well in estimating AGB at the three study sites. For the temperate forest in the Sierra Nevada Mountains, California, USA, R^2^ ranged from 0.79 to 0.8 and RMSE (relRMSE) from 69.69 (35.59%) to 70.71 (36.12%) Mg ha^−1^ for the echo-based model and from 0.76 to 0.78 and 73.84 (37.72%) to 128.20 (65.49%) Mg ha^−1^ for the CHM-based model. For the moist tropical forest on Barro Colorado Island, Panama, the models gave R^2^ ranging between 0.70 and 0.71 and RMSE between 30.08 (12.36%) and 30.32 (12.46) Mg ha^−1^ [between 0.69–0.70 and 30.42 (12.50%) and 61.30 (25.19%) Mg ha^−1^] for the echo-based [CHM-based] models. Finally, for the Atlantic forest in the Sierra do Mar, Brazil, R^2^ was between 0.58–0.69 and RMSE between 37.73 (8.67%) and 39.77 (9.14%) Mg ha^−1^ for the echo-based model, whereas for the CHM R^2^ was between 0.37–0.45 and RMSE between 45.43 (10.44%) and 67.23 (15.45%) Mg ha^−1^.

**Conclusions:**

Metrics derived from the CHM show a higher dependence on point density than metrics derived from the echo-based data model. Despite the median of the differences between metrics derived at different point densities differing significantly from zero, the mean change was close to zero and smaller than the standard deviation except for very low point densities (1 point m^−2^). The application of calibrated models to estimate AGB on metrics derived from thinned datasets resulted in less than 5% error when metrics were derived from the echo-based model. For CHM-based metrics, the same level of error was obtained for point densities higher than 5 points m^−2^. The fact that reducing point density does not introduce significant errors in AGB estimates is important for biomass monitoring and for an effective implementation of climate change mitigation policies such as REDD + due to its implications for the costs of data acquisition. Both data models showed similar capability to estimate AGB when point density was greater than or equal to 5 point m^−2^.

## Background

Forests provide essential ecosystem services at a range of scales and represent a major sink of atmospheric carbon, yet can turn into a significant carbon source due to deforestation and forest degradation. Therefore, identifying the role of forests as carbon sinks or sources is key to understanding the carbon cycle [1]. Likewise, development of precise forest monitoring systems is essential for the effective implementation of climate change mitigation policies such as REDD + (reducing emissions from deforestation and degradation), which require accurate mapping of aboveground biomass (AGB) and its changes.

Numerous studies have proved the ability of LiDAR data to provide accurate estimations of field-measured AGB across different ecosystems [2–5] given its capability of providing detailed 3D measurements of forest structure. Nevertheless, the accuracy of the LiDAR estimation is subject to the accuracy of the field measurements and allometric equations used to derive AGB, which are subsequently used to calibrate LiDAR-based models [6].

Sensor characteristics and flight planning parameters affect LiDAR measurements of the spatial distribution of canopy components and therefore of the vegetation structure metrics derived from them. Similarly, digital elevation models (DEM) and digital surface models (DSM) derived from the LiDAR data are also affected by acquisition parameters. These effects will be propagated to the canopy height model (CHM) obtained by subtracting the DEM from the DSM. The effect of LiDAR survey parameters on the derivation of biophysical properties from airborne LiDAR data has been investigated in different studies. For example [7, 8], concluded that the use of different sensors or variation of flying altitude and pulse repetition frequency (PRF) between different acquisitions result in significant differences of LiDAR metrics sensitive to the vertical distribution of vegetation and canopy density. However, Hopkinson [9] found that laser pulse peak power concentration was the most important factor in the variation of intensity and frequency distribution of returns, although with different effects over short and tall vegetation. Scan angle has also been shown to affect fractional cover (FC) estimates, yet for small scan angles the effect is less evident [7].

In most of these studies, the effect of survey configuration on the LiDAR information was assessed by collecting new data with varying survey parameters. However, variation of acquisition settings like flying height or PRF results in a simultaneous variation of more than one LiDAR parameter like footprint size, point density or pulse power. This makes it difficult to generalize the effect of changing a single parameter on the resulting point cloud. In order to isolate the effect of each survey characteristic on the resulting point clouds and the height estimated from them, Disney et al. [10] simulated different point clouds for different scenarios defined by modifying a single parameter at a time, using a Monte Carlo Ray trace (MCRT) model of canopy scattering. Some of these studies have shown a general increase in the retrieved vegetation height with an increase flying height or reduced PRF [8, 10] whereas the opposite effect has also been reported [7, 9] as a result of a reduction of the pulse energy per unit area.

LiDAR vegetation measures can be represented using two different data models, the echo-based and the CHM raster model. The former represents forest structure by means of 3D point cloud whereas the latter summarizes this information into a raster where each pixel represents the maximum height of the points contained within it. The CHM approach significantly decreases the volume of the data at the expense of loss of information provided. Some studies have evaluated the effect of echo- and CHM-based models on the retrieval of canopy gaps [11] or more recently, on the estimation of AGB [12]. Nevertheless, these studies did not evaluate the impact of varying acquisition parameters on the metrics derived from each data model.

In the context of carbon monitoring, which requires repeated acquisitions at a certain interval, it is likely that each survey will be carried out using different sensors or flight configurations. In addition, in order to maintain cost-efficiency of LiDAR data for REDD + MRV (measuring, reporting and verification), optimum survey configurations should be planned. Point density, along with the footprint size, determines the spatial resolution of LiDAR datasets. It is probably the most important parameter when planning a LiDAR acquisition, with a significant impact in acquisition costs, as it is common to target a minimum point density for the study area in order to maximize spatial coverage. Therefore, the evaluation of the effect of both point density and the data model used on the estimation of AGB becomes an important issue in the MRV process.

This study aims at evaluating the potential of the echo-based and the CHM data models for AGB estimation over three forests across different biomes, and how they are affected by the point density. The specific objectives were to: (1) evaluate the effect of point density on the metrics derived from each data model; (2) evaluate the impact of plot size on the metrics; (3) evaluate the potential of these data models to estimate AGB in different forests with very different vegetation types; and (4) evaluate the impact of point density on the derived empirical models.

## Results

### Effect of point density on the metrics

Tables 1, 2 and 3 show the results of the two-sided Wilcoxon signed rank test for the null hypothesis that there were no statistically significant differences in medians between the metrics derived from the original and the thinned data. In all three sites, the reduction of point density resulted, for most of the metrics, in significant differences between the metrics derived from the original and the thinned datasets. These results were also supported by a two-sided one-sample *t* test of the differences in means between the original and the thinned datasets (results not shown). Point density reduction had larger impact on the metrics derived from the CHM than on those derived from the echo-based model. The effect of point density on the metrics also generally showed similar behavior for the different plot sizes tested, from 0.09 to 1 ha. Although similar patterns were observed at the three study sites, some differences exist among them, reflecting differences in their vegetation structure. For instance, the area under the canopy waveform (AUCW) obtained from the echo-based model showed significant differences in the Sierra Nevada Mountains in California (SNM herein after) and on Barro Colorado Island, Panama (BCI herein after), whereas in the Sierra do Mar in Brazil (SdM herein after) differences were not statistically significant. Similarly, while differences in fractional cover (FC) or the standard deviation of the height (StdH) were significantly different from zero for any point density or data model in SNM, in BCI and SdM the differences were only significant for the lowest point density (1 point m^−2^).

**Table 1:** Two-sided Wilcoxon signed rank test results of the point density effect on LiDAR metrics for the Sierra Nevada Mountains study site

Sierra Nevada Mountains, California																					
Plot size (ha)	Point density (points m^−2^)	AUCW		CV		FC		Max-H		Mean-H		P25H		P50H		P75H		P90H		StdH	
		E-B	CHM	E-B	CHM	E-B	CHM	E-B	CHM	E-B	CHM	E-B	CHM	E-B	CHM	E-B	CHM	E-B	CHM	E-B	CHM
0.09	Orig-10p	✓	✓	–	✓	✓	✓	–	–	✓	✓	✓	✓	✓	✓	✓	✓	✓	✓	✓	–
	Orig-5p	✓	✓	–	✓	✓	✓	✓	✓	–	✓	–	✓	–	✓	✓	✓	✓	✓	–	–
	Orig-1p	✓	✓	–	✓	✓	✓	✓	✓	–	✓	–	✓	–	✓	–	✓	–	✓	–	✓
0.25	Orig-10p	✓	✓	–	✓	✓	✓	–	–	✓	✓	✓	✓	✓	✓	✓	✓	✓	✓	✓	–
	Orig-5p	✓	✓	–	✓	✓	✓	✓	✓	–	✓	–	✓	–	✓	✓	✓	–	✓	✓	✓
	Orig-1p	✓	✓	–	✓	–	✓	✓	✓	–	✓	–	✓	–	✓	–	✓	–	✓	–	✓
0.5	Orig-10p	✓	✓	–	✓	✓	✓	–	–	✓	✓	✓	✓	✓	✓	✓	✓	✓	✓	✓	✓
	Orig-5p	✓	✓	–	✓	✓	✓	✓	✓	–	✓	–	✓	–	✓	–	✓	✓	✓	✓	✓
	Orig-1p	–	✓	–	✓	✓	✓	✓	✓	–	✓	✓	✓	–	✓	–	✓	–	✓	–	✓
1.00	Orig-10p	✓	✓	–	✓	✓	✓	–	–	✓	✓	✓	✓	✓	✓	✓	✓	✓	✓	✓	✓
	Orig-5p	✓	✓	–	✓	✓	✓	✓	✓	–	✓	–	✓	–	✓	✓	✓	✓	✓	✓	✓
	Orig-1p	✓	✓	✓	✓	✓	✓	✓	✓	–	✓	✓	✓	–	✓	–	✓	–	✓	✓	✓

– Indicates no significant differences, ✓ indicates significant differences (significance level: 5%), *E-B* echo-based data model, *CHM* canopy height model, *AUCW* area under canopy waveform, *CV* coefficient of variation, *FC* fractional cover, *Max-H* maximum canopy height, *Mean-H* mean canopy height, *P25H* 25th height percentile, *P50H* 50th height percentile (median height), *P75H* 75th height percentile, *P90H* 90th height percentile, *StdH* standard deviation of the height, *Orig* point density of the original dataset, *10p* point density reduced to 10 p m^−2^, *5p* point density reduced to 5 p m^−2^, *1p* point density reduced to 1 p m^−2^

**Table 2:** Two-sided Wilcoxon signed rank test results of the point density effect on LiDAR metrics for the Barro Colorado Island study site

Barro Colorado Island, Panama																					
Plot size (ha)	Point density (points m^−2^)	AUCW		CV		FC		Max-H		MeanH		P25H		P50H		P75H		P90H		StdH	
		E-B	CHM	E-B	CHM	E-B	CHM	E-B	CHM	E-B	CHM	E-B	CHM	E-B	CHM	E-B	CHM	E-B	CHM	E-B	CHM
0.09	Orig-5p	–	✓	–	✓	–	✓	–	–	–	✓	–	✓	–	✓	–	✓	–	✓	–	✓
	Orig-1p	✓	✓	–	✓	✓	✓	✓	✓	–	✓	–	✓	–	✓	–	✓	–	✓	–	✓
0.25	Orig-5p	✓	–	✓	✓	–	✓	–	–	–	✓	–	✓	–	✓	✓	✓	–	✓	✓	✓
	Orig-1p	✓	✓	–	✓	✓	✓	✓	✓	–	✓	–	✓	–	✓	–	✓	–	–	–	✓
0.50	Orig-5p	✓	✓	–	✓	–	✓	–	–	–	✓	–	✓	–	✓	–	✓	–	✓	✓	✓
	Orig-1p	✓	✓	–	✓	✓	✓	✓	✓	✓	✓	–	✓	–	✓	✓	✓	✓	✓	–	✓
1.00	Orig-5p	✓	✓	–	✓	–	✓	–	–	✓	✓	–	✓	✓	✓	–	✓	–	✓	✓	✓
	Orig-1p	✓	✓	–	✓	✓	✓	✓	✓	✓	✓	–	✓	–	✓	✓	✓	✓	✓	–	✓

– indicates no significant differences, ✓ indicates significant differences (significance level: 5%), *E-B* echo-based data model, *CHM* canopy height model, *AUCW* area under canopy waveform, *CV* coefficient of Variation, *FC* fractional cover, *Max-H* maximum canopy height, *Mean-H* mean canopy height, *P25H* 25th height percentile, *P50H* 50th height percentile (median height), *P75H* 75th height percentile, *P90H* 90th height percentile, *StdH* standard deviation of the height, *Orig* point density of the original dataset, *5p* point density reduced to 5 p m^−2^, *1p* point density reduced to 1 p m^−2^

**Table 3:** Two-sided Wilcoxon signed rank test results of the point density effect on LiDAR metrics for the Serra do Mar study site

Serra do Mar, Brazil																					
Plot size (ha)	Point density (points m^−2^)	AUCW		CV		FC		Max-H		MeanH		P25H		P50H		P75H		P90H		StdH	
		E-B	CHM	E-B	CHM	E-B	CHM	E-B	CHM	E-B	CHM	E-B	CHM	E-B	CHM	E-B	CHM	E-B	CHM	E-B	CHM
0.09	Orig-10p	–	–	–	✓	–	–	–	–	–	✓	–	✓	–	✓	–	✓	–	✓	–	✓
	Orig-5p	–	–	–	✓	–	–	–	–	–	✓	–	✓	✓	✓	–	✓	✓	✓	–	✓
	Orig-1p	–	✓	✓	✓	✓	✓	✓	✓	✓	✓	–	✓	✓	✓	✓	✓	✓	✓	–	✓
0.25	Orig-10p	–	–	–	✓	–	✓	–	–	–	✓	–	✓	–	✓	–	✓	–	–	–	✓
	Orig-5p	–	✓	–	✓	–	✓	–	–	–	✓	–	✓	–	✓	✓	✓	–	✓	–	✓
	Orig-1p	–	✓	✓	✓	✓	✓	✓	✓	✓	✓	–	✓	✓	✓	✓	✓	✓	✓	–	✓
0.50	Orig-10p	–	✓	–	✓	–	–	–	–	–	✓	–	✓	–	✓	–	✓	–	✓	–	✓
	Orig-5p	–	✓	–	✓	–	✓	–	–	✓	✓	✓	✓	–	✓	✓	✓	✓	✓	–	✓
	Orig-1p	–	✓	✓	✓	✓	✓	–	–	✓	✓	✓	✓	✓	✓	✓	✓	–	✓	–	✓
1.00	Orig-10p	–	✓	–	✓	–	✓	✓	✓	–	✓	–	✓	–	✓	–	✓	–	✓	–	✓
	Orig-5p	–	✓	–	–	–	✓	✓	✓	–	✓	–	✓	–	✓	✓	✓	✓	✓	–	✓
	Orig-1p	–	✓	–	✓	✓	✓	–	–	✓	✓	–	✓	✓	✓	✓	✓	–	✓	–	✓

– indicates no significant differences, ✓ indicates significant differences (significance level: 5%), *E-B* echo-based data model, CHM canopy height model, *AUCW* area under canopy waveform, *CV* coefficient of variation, *FC* fractional cover, *Max-H* maximum canopy height, *Mean-H* mean canopy height, *P25H* 25th height percentile, *P50H* 50th height percentile (median height), *P75H* 75th height percentile, *P90H* 90th height percentile, *StdH* standard deviation of the height, *Orig* point density of the original dataset, *10p* point density reduced to 10 p m^−2^, *5p* point density reduced to 5 p m^−2^, *1p* point density reduced to 1 p m^−2^

Although the statistical test resulted in significant differences for the metrics derived at different point densities, the magnitude of these differences was generally very low. In all three sites, canopy height values decreased as the point density was reduced. The mean difference between the maximum height estimated from the highest point density and the thinned data was negligible, except for the lowest point density (1 point m^−2^), for which it could be larger than 1 m. In addition, in most cases the standard deviation of the differences was larger than the mean. Thus, the mean differences (±standard deviation) in maximum canopy height ranged between −0.02 m (±0.20 m) and 1.16 m (±0.87 m) in SNM, −0.03 m (±0.66 m) and 0.84 m (±1.73 m) in BCI and 0 (±0.94 m) and 0.97 m (±1.37 m) in SdM. The same trends were observed regardless of the data model used, although differences from the CHM-derived metrics were larger. The same pattern was observed for other metrics related to the vertical distribution of vegetation (mean and percentiles of the height) derived from the echo-based model. Differences ranged between −0.09 m (±0.10 m) and 0.06 m (±0.20 m) in SNM, between −0.08 m (±0.37 m) and 0.11 m (±0.48 m) in BCI and between −0.52 m (±0.56 m) and 0.96 m (±0.66 m) in SdM. Height metrics derived from the CHM were more affected by point density with differences ranging between 0.29 m (±0.23 m) and 5.09 m (±3.49 m) in SNM, 0.25 (±0.50 m) and 5.19 m (±1.93 m) in BCI and 0.14 m (±0.12 m) and 4.82 m (±1.20 m) in SdM. These differences were statistically significant and unlike for the metrics derived from the echo-based model, the standard deviation was smaller than the mean. In all cases, the largest differences were attained at the lowest point density (1 point m^−2^). In the case of the coefficient of variation of the height, differences were generally not significant for all three sites when it was derived from the echo-based model but became significant when derived from the CHM-based model. In the case of the standard deviation of the height, different behavior was observed for each study site, with significant differences observed in SNM but not in SdM. When the metric was derived from the CHM, the differences were significant at all three sites. Moreover, while the differences in the standard deviation of the height were less than 15 cm when derived from the echo-based model, they were larger than 1 m when derived from the CHM in SNM and BCI. Regarding the AUCW, smaller values were obtained as the point density was reduced, particularly when derived from the CHM. Finally, in the case of FC, differences were less than 2% in all three sites when derived from the echo-based model, despite being statistically significant for the SNM study site. Slightly larger differences were obtained when FC was derived from the CHM, with values reaching 5% in SNM, 14% in BCI and 2% for the SdM.

**Fig. 1:**
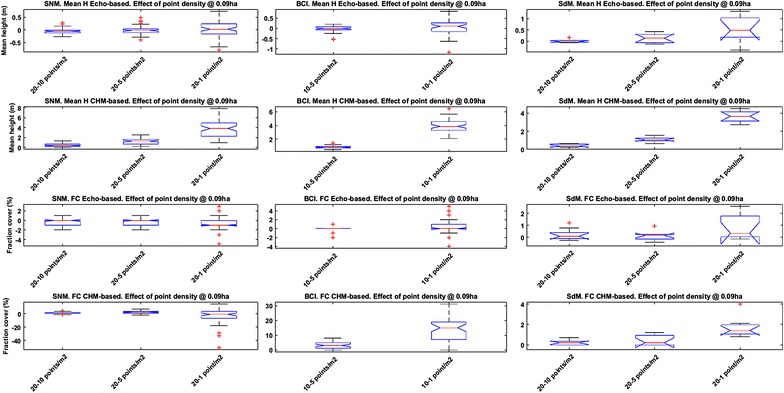
Boxplots of mean height and FC for each study site and point density. *Left column* Sierra Nevada Mountains; *central column* Barro Colorado Island; *right column* Serra do Mar (SdM). *Top row* echo-based mean canopy height; *second row* CHM-based mean canopy height; *third row* echo-based FC; *bottom row* CHM-based FC

The boxplots in Fig. 1 show a summary of the variation of mean canopy height and FC subsequently used to model AGB, derived from each data model at each study site as a function of point density. These variables were used to model AGB from the LiDAR data.

### Effect of plot size on the metrics

The effect of structural variability associated with the plot size varied among the study sites and the data models used. Variables like AUCW, coefficient of variation and StdH, showed different behavior in each study site. The same pattern of the effect of plot size on the metrics was observed for the different point densities evaluated (Tables 4, 5 and 6). FC and height related metrics, except maximum height and P25H, did not show statistically significant differences (p value >0.05). Maximum canopy height showed significant differences at all sites as it could be expected with absolute differences ranging between 3.15 m (±3.55 m) and 8.13 (±4.54 m); 1.67 m (±3.26 m) and 6.64 m (±6.19 m); and 2.57 m (±2.08 m) and 7.24 m (±0.44 m) for SNM, BCI and SdM, respectively. P25H showed different behavior for SNM than for BCI and SdM, which could be a result of a more open canopy. Whereas for SNM the differences ranged between 0.45 m (±1.78 m) and 0.90 m (±0.28 m), for BCI the range was from −0.05 m (±1.42 m) to −0.46 (±3.60 m) and for SdM they spanned from −0.01 m (±1.60 m) to −0.48 m (±1.49 m). A monotonic effect of plot size on the metrics was observed at all point densities, i.e. an increase or decrease as the plot size varied, regardless of the data model used or the study site.

**Table 4:** Two-sided Wilcoxon signed rank test results of the plot size effect on LiDAR metrics for the Sierra Nevada Mountains study site

Sierra Nevada Mountains, California																					
Point density (points m^−2^)	Plot size (ha)	AUCW		CV		FC		Max-H		MeanH		P25H		P50H		P75H		P90H		StdH	
		E-B	CHM	E-B	CHM	E-B	CHM	E-B	CHM	E-B	CHM	E-B	CHM	E-B	CHM	E-B	CHM	E-B	CHM	E-B	CHM
Original	0.09–0.25	–	–	✓	✓	–	–	✓	✓	–	–	–	✓	–	–	–	–	–	–	✓	✓
	0.09–0.50	–	–	✓	✓	–	–	✓	✓	–	–	✓	✓	–	–	–	–	–	–	✓	✓
	0.09–1.00	✓	✓	✓	✓	✓	–	✓	✓	–	✓	✓	✓	–	✓	–	–	✓	–	✓	✓
10 points	0.09–0.25	–	–	✓	✓	–	–	✓	✓	–	–	✓	✓	–	–	–	–	–	–	✓	✓
	0.09–0.50	–	✓	✓	✓	–	–	✓	✓	–	–	✓	✓	–	–	–	–	–	–	✓	✓
	0.09–1.00	✓	✓	✓	✓	✓	–	✓	✓	–	✓	✓	✓	–	✓	–	–	✓	–	✓	✓
5 points	0.09–0.25	–	–	✓	✓	–	–	✓	✓	–	–	✓	✓	–	–	–	–	–	–	✓	✓
	0.09–0.50	–	✓	✓	✓	–	–	✓	✓	–	–	✓	✓	–	–	–	–	–	–	✓	✓
	0.09–1.00	✓	✓	✓	✓	✓	–	✓	✓	–	✓	✓	✓	–	✓	–	–	✓	–	✓	✓
1 points	0.09–0.25	–	✓	✓	✓	–	–	✓	✓	–	–	–	✓	–	–	–	–	–	–	✓	–
	0.09–0.50	–	✓	✓	✓	–	–	✓	✓	–	–	✓	✓	–	–	–	–	–	–	✓	–
	0.09–1.00	✓	✓	✓	✓	✓	–	✓	✓	–	✓	✓	✓	–	✓	–	–	✓	–	✓	✓

The reference plot size for the comparison was that used for the field plot measurements (0.09 ha)

– indicates no significant differences, ✓ indicates significant differences (significance level: 5%), *E-B* echo-based data model, *CHM* canopy height model, *AUCW* area under canopy waveform, *CV* coefficient of variation, *FC* fractional cover, *Max-H* maximum canopy height, *Mean-H* mean canopy height, *P25H* 25th height percentile, *P50H* 50th height percentile (median height), *P75H* 75th height percentile, *P90H* 90th height percentile, *StdH* standard deviation of the height

**Table 5:** Two-sided Wilcoxon signed rank test results of the plot size effect on LiDAR metrics for the Barro Colorado Island study site

Barro Colorado Island, Panama																					
Point density (points m^−2^)	Plot size (ha)	AUCW		CV		FC		Max-H		MeanH		P25H		P50H		P75H		P90H		StdH	
		E-B	CHM	E-B	CHM	E-B	CHM	E-B	CHM	E-B	CHM	E-B	CHM	E-B	CHM	E-B	CHM	E-B	CHM	E-B	CHM
Original	1.00–0.09	✓	✓	✓	✓	–	–	✓	✓	–	–	–	–	–	–	–	–	✓	✓	✓	✓
	1.00–0.25	✓	✓	–	–	–	–	✓	✓	–	–	–	–	–	–	–	–	–	–	✓	✓
	1.00–0.50	✓	✓	–	–	–	–	✓	✓	–	–	–	–	–	–	–	–	–	–	–	–
5 points	1.00–0.09	✓	✓	✓	✓	–	–	✓	✓	–	–	–	–	–	–	–	–	✓	✓	✓	✓
	1.00–0.25	✓	✓	–	–	–	–	✓	✓	–	–	–	–	–	–	–	–	–	–	✓	✓
	1.00–0.50	✓	✓	–	–	–	–	✓	✓	–	–	–	–	–	–	–	–	–	–	–	–
1 points	1.00–0.09	✓	✓	✓	✓	–	–	✓	✓	–	–	–	–	–	–	–	–	✓	✓	✓	–
	1.00–0.25	✓	✓	–	–	–	–	✓	✓	–	–	–	–	–	–	–	–	–	–	✓	–
	1.00–0.50	✓	✓	–	–	–	–	✓	✓	–	–	–	–	–	–	–	–	–	–	–	–

The reference plot size for the comparison was that used for the field plot measurements (1.00 ha)

– indicates no significant differences, ✓ indicates significant differences (significance level: 5%), *E-B* echo-based data model, *CHM* canopy height model, *AUCW* area under canopy waveform, *CV* coefficient of variation, *FC* fractional cover, *Max-H* maximum canopy height, *Mean-H* mean canopy height, *P25H* 25th height percentile, *P50H* 50th height percentile (median height), *P75H* 75th height percentile, *P90H* 90th height percentile, *StdH* standard deviation of the height

**Table 6:** Two-sided Wilcoxon signed rank test results of the plot size effect on LiDAR metrics for the Serra do Mar study site

Serra do Mar, Brazil																					
Point density (points m^−2^)	Plot size (ha)	AUCW		CV		FC		Max-H		MeanH		P25H		P50H		P75H		P90H		StdH	
		E-B	CHM	E-B	CHM	E-B	CHM	E-B	CHM	E-B	CHM	E-B	CHM	E-B	CHM	E-B	CHM	E-B	CHM	E-B	CHM
Original	1.00–0.09	✓	–	–	–	–	–	✓	✓	–	–	–	–	–	–	–	–	–	–	–	✓
	1.00–0.25	–	–	–	–	–	–	✓	✓	–	–	–	–	–	–	–	–	–	–	–	–
	1.00–0.50	–	–	–	–	–	–	✓	✓	–	–	–	–	–	–	–	–	–	–	–	–
10 points	1.00–0.09	✓	–	–	–	–	–	✓	✓	–	–	–	–	–	–	–	–	–	–	–	–
	1.00–0.25	✓	–	–	–	–	–	✓	✓	–	–	–	–	–	–	–	–	–	–	–	–
	1.00–0.50	–	–	–	–	–	–	✓	✓	–	–	–	–	–	–	–	–	–	–	–	–
5 points	1.00–0.09	✓	✓	–	–	–	–	✓	✓	–	–	–	–	–	–	–	–	–	–	–	✓
	1.00–0.25	✓	–	–	–	–	–	✓	✓	–	–	–	–	–	–	–	–	–	–	–	–
	1.00–0.50	–	–	–	–	–	–	✓	✓	–	–	–	–	–	–	–	–	–	–	–	–
1 points	1.00–0.09	✓	–	–	–	–	–	✓	✓	–	–	–	–	–	–	–	–	–	–	–	–
	1.00–0.25	✓	–	–	–	–	–	✓	✓	–	–	–	–	–	–	–	–	–	–	–	–
	1.00–0.50	–	–	–	–	–	–	✓	✓	–	–	–	–	–	–	–	–	–	–	–	–

The reference plot size for the comparison was that used for the field plot measurements (1.00 ha)

–indicates no significant differences, ✓ indicates significant differences (significance level: 5%), *E-B* echo-based data model, *CHM* canopy height model, *AUCW* area under canopy waveform, *CV* coefficient of variation, *FC* fractional cover, *Max-H* maximum canopy height, *Mean-H* mean canopy height, *P25H* 25th height percentile, *P50H* 50th height percentile (median height), *P75H* 75th height percentile, *P90H* 90th height percentile, *StdH* standard deviation of the height

The CHM-derived metrics generally showed a greater dependence on the plot size than the echo—based metrics, especially for the SNM. Although for BCI and SdM the mean differences of the metrics were similar, with the exception of AUCW and P25H, the standard deviation for the metrics derived from the CHM was higher for all metrics and study sites.

**Fig. 2:**
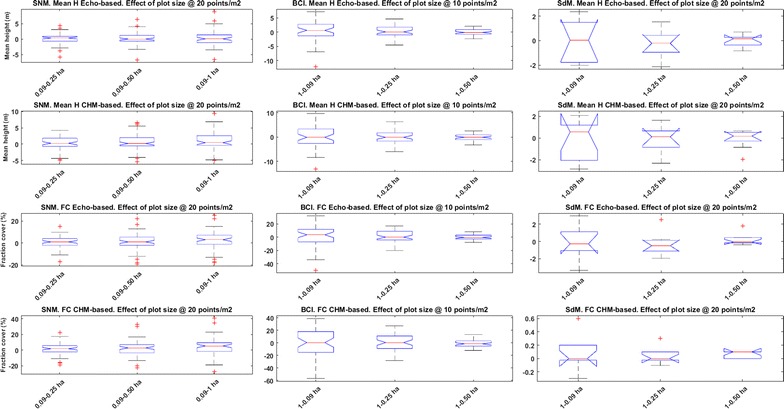
Boxplots of the differences in mean height and FC for each study site and plot size. The reference data for the comparison were the original point density and the plot size used for field measurements. *Left column* Sierra Nevada Mountains; *central column* Barro Colorado Island; *right column* Serra do Mar. *Top row* Echo-based mean canopy height; *second row* CHM-based mean canopy height; *third row* Echo-based FC; *bottom row* CHM-based FC

The boxplots in Fig. 2 show a summary of the variation of mean canopy height and FC derived from each data model at each study site as a function of plot size.

### Aboveground biomass modeling

Table 7 presents the results of the power models adjusted to estimate AGB for each study site and data model. It also presents the effect of point density on the model derived at the original point density. Both data models performed similarly in all study sites and no effect of point density was observed for the echo-based model. This was expected due to the small changes observed in mean height and FC when the metrics were derived at different point densities. Although differences between the AGB estimates from the thinned datasets were statistically significant (p value <0.05), except for SdM, the largest error for SNM was only 4% of the mean AGB derived from the model trained with the highest point density. For BCI and SdM the largest errors represented less than 1 and 5%, respectively. In all three sites, the CHM-based model showed a remarkable decrease in performance when applied to the lowest point density (1 point m^−2^). This effect was not reflected in terms of R^2^ but in the RMSE. Moreover, the largest error represented up to 48, 23 and 15% of the mean AGB derived from the model trained with the highest point density for SNM, BCI and SdM, respectively. The inclusion of FC in the model slightly improved results in SNM and SdM but had no effect in BCI.

**Table 7:** Model (echo-based and CHM) evaluation for each study site and power model fitted

Study site	Data model	Model	Parameters	Point density (points m^−2^)	R^2^	RMSE (Mg ha^−1^)	relRMSE(%)
Sierra Nevada Mountains	Echo	$\hat{y} = \alpha X^{\beta }$	α = 2.75; β = 1.52	20	0.70	85.15	43.50
				10	0.70	85.00	43.42
				5	0.71	84.13	42.98
				1	0.70	85.10	43.47
	CHM		α = 11.72; β = 1.07	20	0.70	84.87	43.36
				10	0.70	87.72	44.81
				5	0.71	93.90	47.97
				1	0.74	120.77	61.69
	Echo	$\hat{y} = \alpha x_{1}^{\beta } x_{2}^{\gamma }$	α = 11.50; β = 1.20; γ = 0.88	20	0.79	70.67	36.10
				10	0.79	70.71	36.12
				5	0.80	69.66	35.59
				1	0.79	70.15	35.83
	CHM		α = 4.22; β = 1.39; γ = −0.62	20	0.76	73.84	37.72
				10	0.77	76.98	39.33
				5	0.78	85.43	43.64
				1	0.78	128.20	65.49
Barro Colorado Island	Echo	$\hat{y} = \alpha X^{\beta }$	α = 1.80; β = 1.61	10	0.71	30.08	12.36
				5	0.71	30.12	12.38
				1	0.70	30.32	12.46
	CHM		α = 2.07; β = 1.49	10	0.70	30.42	12.50
				5	0.70	31.78	13.06
				1	0.69	61.30	25.19
	Echo	$\hat{y} = \alpha x_{1}^{\beta } x_{2}^{\gamma }$	α = 9.24; β = 1.12; γ = 0.11	10	0.71	30.22	12.42
				5	0.71	30.25	12.43
				1	0.70	30.37	12.48
	CHM		α = 12.32; β = 0.97; γ = 0.13	10	0.69	31.00	12.74
				5	0.70	31.75	13.04
				1	0.70	55.93	22.98
Serra do Mar	Echo	$\hat{y} = \alpha X^{\beta }$	α = 2.69 (3.08); β = 1.88 (0.26)	20	0.44	45.71	10.50
				10	0.40	48.02	11.03
				5	0.44	46.02	10.57
				1	0.58	44.42	10.21
	CHM		α = 1.68 (2.47); β = 2.00 (0.33)	20	0.45	45.47	10.45
				10	0.40	50.63	11.63
				5	0.40	63.36	14.56
				1	0.46	136.39	31.34
	Echo	$\hat{y} = \alpha x_{1}^{\beta } x_{2}^{\gamma }$	α = 0.68 (1.43); β = 2.55 (0.42); γ = −3.3 (1.08)	20	0.62	37.73	8.67
				10	0.58	39.77	9.14
				5	0.59	39.26	9.02
				1	0.69	38.93	8.94
	CHM		α = 2.70 (6.60); β = 2.14 (0.48); γ = −16.79 (16.34)	20	0.45	45.43	10.44
				10	0.38	48.90	11.24
				5	0.40	49.53	11.38
				1	0.37	67.23	15.45

Model parameters are presented for each model. For Sierra Nevada Mountains and Barro Colorado Island 70% of the plots were used for calibration and 30% for independent validation. For Serra do Mar due to the small size of the sample a jackknife approach was used instead

Standard deviation of the parameters is presented in brackets

**Fig. 3:**
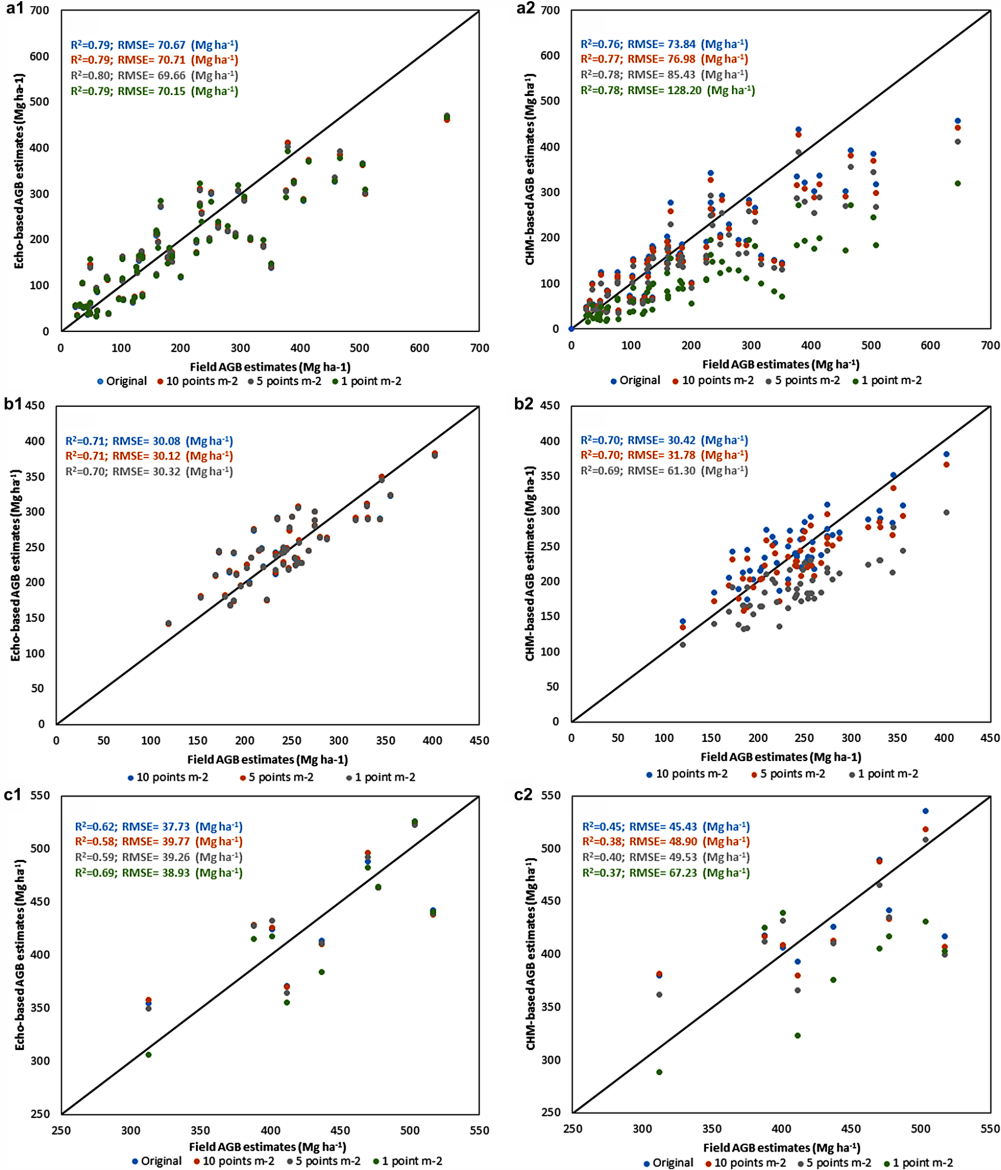
Scatterplots of LiDAR vs field AGB estimates. **a** Sierra Nevada Mountains; **b** Barro Colorado Island; **c** Sierra do Mar. (1) Echo-basedmodel; (2) CHM-based model. Models were calibrated using the highest point density available at each study site. The effect of point density of the estimate was evaluated by applying the calibrated model to the thinned data. The *solid line* represents the 1:1 line

Figure 3 shows the scatter plot of the estimated AGB from the different models and resolutions compared to the field measurements. Points almost overlap when the model is calibrated using echo-derived metrics whereas higher discrepancies are observed in the CHM-based models. This trend is observed in the three study sites.

## Discussion

### Effect of point density on the metrics

Height metrics derived from the echo-based model were less affected by a reduction in point density than those derived from the CHM, particularly when the point density was reduced to 1 point m^−2^. This is explained by the fact that the metrics from the point cloud were computed considering all canopy returns (h > 2 m); whereas for the CHM the height of each pixel used to compute them represented the highest return within it. The effect of interpolation on the generation of the CHM also contributes to the effect of point density on the metrics derived from the CHM. We found a decrease in vegetation height estimated from LiDAR with decreasing point density, as a result of a lower probability of laser pulses hitting the top of the canopy as well as fewer of them penetrating to the ground, which affects the DEM generation. This agrees with the results obtained by Disney et al. [10]. The reduction of mean CHM height with point density observed in SdM was also reported by Leitold et al. [13]. for the same study area. However, they found greater differences, which is probably due to the fact that we applied different filtering algorithms to classify ground returns for the thinned data.

The coefficient of variation did not show any significant differences for the echo-based model but did for the CHM. This could also be a result of the different consideration of vegetation data in the computation of the metrics for each data model, i.e. returns above 2 m for the echo-based model vs all pixels for the CHM-based metrics. For the AUCW forest structure seemed to be a determinant factor in the variation of the metric. In BCI it presented values 2–3 times larger than in the other two study sites and the reduction of point density caused a larger impact on the obtained values. The CHM model was more affected than the echo-based model since it only captures the variation of the upper canopy.

Although the statistical tests indicated that the median differences of the metrics derived from different point densities significantly differed from zero, the mean change was very low in most cases except when the point density decreased to 1 point m^−2^. Besides, the standard deviation of the change was larger than the mean itself and therefore, the variation in point density did not result in a significant trend of variation in the metrics.

Several authors [7, 8, 10] have pointed out the difficulty in comparing results from different study sites, sensor characteristics and survey configurations. However, our results using different sensors and flight specifications over remarkably different forest types, showed similar results, giving confidence in the robustness of our results.

It should be borne in mind that we simply reduced the point density by randomly removing LiDAR returns. In this way, we only changed the number of returns available to compute height and vegetation density metrics but other effects associated to the variation of flying parameters like footprint size or pulse penetration were not altered. However, in an operational scenario, point density is reduced by changing either PRF or flight elevation, which makes other parameters like footprint size co vary with point density. The different effects of flight and sensor configurations on different echo categories [7, 9, 14] could also influence some of the metrics derived to account for the vertical distribution of vegetation. Despite the limitations of the method applied for point density reduction, our results agree with other studies collecting different datasets under varying data acquisition configurations.

### Effect of plot size on the metrics

Variation of plot size showed different effects for each study site for most of the metrics. Disney et al. [10] showed the impact of vertical distribution of vegetation on the distribution of returns. Varying the plot size changed the scale at which the structural patterns were measured. Our results showed that metrics describing the canopy height (Hmax) and the vertical distribution of canopy material through the whole canopy such as AUCW, P25H, CV of the height or StdH were more affected than metrics representing mean height or other height-related percentiles. This effect also depended on both the data model and the study site. A significant impact of different canopy structures, stratified by age classes and vegetation type, on canopy height was also described in other studies [9, 10, 15]. As expected, maximum canopy height showed significant differences since this metric represents a single measurement for a given plot. The fact that metrics like mean height or height percentiles were not affected by the plot size indicates certain homogeneity in the structure of our study sites at different scales. In general terms, the differences in height percentiles or mean height were below 1 m and smaller than the standard deviation of the change when derived from the echo-based model, whereas slightly higher differences were observed when derived from the CHM. The same was observed in FC, with changes smaller than 2%.

### Aboveground biomass modeling

The two data models used performed almost equally in estimating AGB, which agrees with the results obtained by Chirici et al. [12] who found that the data model had no significant impact on the results. Table 7 shows that R^2^ and RMSE were almost identical regardless of the data model used. BCI and SNM showed similar R^2^ values but the RMSE for SNM was much higher. This is related to the smaller plot size of 0.09 ha for SNM compared to 1 ha for BCI. Mascaro et al. [16] showed a decrease of 38% in the uncertainty of LiDAR based carbon estimates in tropical areas when field plots were scaled from 0.36 to 1 ha. Similarly, Frazer et al. [15] reported an increase in model accuracy as plots increased from 10 to 25 m radius. An increase in plot size reduces the effect of co-registration errors between field and LiDAR data, reduces the perimeter-to-area ratio, i.e. the edge effect, and reduces sample variance [15], thus increasing model accuracy. Nevertheless, forest structure plays a significant role in the impact of plot size on model accuracy, with different optimum sizes found for tropical and temperate coniferous forests [15, 16]. As for SdM, the plot size was also 1 ha yet the R^2^ values obtained were generally low, around 0.4–0.5. By including the fractional cover into the echo-based model, we improved its performance from R^2^ = 0.44 and RMSE of 45.71 Mg ha^−1^ to an R^2^ = 0.6 and RMSE of 37.73 Mg ha^−1^. However, for the CHM-based model the inclusion of FC did not improve results in terms of R^2^ but reduced the RMSE; especially at 1 point m^−2^ for which the RMSE was reduced to half of the value obtained using only the mean height. Similarly, for the SNM study site, the inclusion of FC into the model also resulted in an improvement of the model by nearly 10% in R^2^ and 8% in RMSE. Several studies have shown that FC can improve AGB estimation from LiDAR data, particularly for heterogeneous forests [2, 17, 18].

For SdM, we obtained better performance when the model was applied to the lowest point density. However, the small sample size (n = 9) prevented us from drawing conclusions and further research is needed. Our results based on the mean canopy height are similar to those obtained by Leitold et al. [13] for the same study area, who obtained an R^2^ = 0.43. In that study, they pointed out a significant impact of point density on mean canopy height which translated to errors in AGB estimations. For the same study area and datasets, we only found significant differences in the estimates for the lowest point density. Nevertheless, this agrees with Leitold et al.’s results, who found that the error in the estimated biomass from the thinned datasets only were greater than the model error when the point density was lower than 4 points m^−2^.

Although the AGB estimates obtained by applying the calibrated model to the metrics derived from the thinned data significantly differed from those obtained from the original data, the errors represented less than 5% in all three sites. Therefore, the model can be applied to lower density data without a significant loss in accuracy. Larger discrepancies were found when the metrics were derived from the CHM. In this case, the errors were bounded by 5% when the point density was reduced to 5 point m^−2^ in BCI and SdM, whereas in SNM this error was obtained only at 10 points m^−2^.

The application of the CHM-based model calibrated at the original point density to metrics derived at the lowest point density showed a notable decrease in performance, for all study sites, as result of the impact of point density on the mean canopy height derived from the CHM. Nevertheless, almost identical accuracy was obtained when specific models were trained at the lowest resolution (SNM: R^2^ = 0.79, RMSE = 69.57, relRMSE(%) = 35.54; BCI: R^2^ = 0.69, RMSE = 30.82, relRMSE(%) = 12.66; SdM:R^2^ = 0.62, RMSE = 37.60, relRMSE(%) = 8.64).

## Conclusions

This study has analyzed the effects of point density and LiDAR data model on AGB estimates. Both data models, echo-based and CHM, showed an almost identical performance across different ecosystems.

Metrics derived from different point densities were significantly different; however, the magnitude of the mean differences were very small, with values of a few centimeters when the point density was higher than or equal to 5 points m^−2^. A larger impact of point density was observed in metrics derived from the CHM. Despite the significant differences among metrics derived from different point densities, the fact that their effect on AGB estimates was less than 5% is important for biomass monitoring and for an effective implementation of climate change mitigation policies such as REDD+. Usually, LiDAR acquisitions are configured to achieve a given point density, which has important economic implications. For instance, we showed that using echo-based metrics point densities as low as 1 point m^−2^ are as effective as very high density datasets. Nevertheless, when CHM-based metrics were used we found that the accuracy of the AGB models was significantly reduced for point densities below 5 point m^−2^. The ability of low point density datasets to estimate AGB represents a cost reduction for AGB mapping that would allow covering larger areas to better capture ABG spatial variability at the landscape level.

Although our point thinning approach did not recreate realistic distributions of returns and did not consider the covarying effect of return parameters when survey parameters are changed, our results on metrics variation patterns agree with other studies that actually varied the surveying parameters. Also, the consistency of results over varying biomes and sensor characteristics reinforce our conclusions.

## Methods

### Study sites

We used three study sites: The first site was the SNM in California. The area was affected by a megafire in 17 August 2013. Our LiDAR data covered the burned area (section datasets) plus a 2 km buffer of unburned vegetation. We restricted our analyses to the unburned buffer. The area presents a rough topography with elevations ranging from 60 to 2400 m, and mean slopes of 17.5%. Vegetation of this area is characterized by chaparral and foothill-oak woodland habitat, conifer forests in the lower montane zone and mixed conifer forests in higher elevation areas. A more detailed description of the study site can be found in Casas et al. [19].

The BCI study site is located in Panama and it is covered by an old-growth moist tropical forest part of the Barro Colorado Nature Monument, which is a protected national reserve. The island receives approximately 2636 mm of annual precipitation and has a four-month dry season between January and April when 10% of the canopy species lose their leaves [20]. Here, we focus on a 50 ha forest inventory plot managed by the Center for Tropical Forest Science (CTFS) that contains some of the largest trees on the Island (up to 54 m).Although most of the study site is located in the main plateau of BCI (<10° in slope), its south-eastern edge shows slopes that exceed 30–40° [21]. Additional detail scan be found in Condit et al. [20].

The third study site is the Sao Paulo State Park of “Serra do Mar” (SdM) in southeast Brazil (23°34′S and 45°02′W; 23°17′S and 45°11′W). The area is covered by the dense vegetation of the Atlantic Forest, under a complex terrain with a large elevation gradient 0–1200 m. The predominant vegetation type is tropical moist evergreen forest [22] or lowland to lower montane rainforest [23]. The average annual rainfall is approximately 3000 mm (with the lowest precipitation in June: 87 mm) and the yearly average temperature is 22 °C [24]. More detailed information of this site can be found in Leitold et al. [13] and Vieira et al. [24].

### Datasets

#### LiDAR data

For the SNM the LiDAR data were collected on November 2013 by the National Center for Airborne Laser Mapping (NCALM) using an Optech Gemini Airborne Laser Terrain Mapper (ALTM) instrument that recorded up to four returns per pulse. The average point density was approximately 20 points m^−2^. A 1 m digital elevation model (DEM) was also provided along with the point cloud, which was used to normalize the height of each return. Further information can be found in Garcia et al. [25].

For BCI the LiDAR data were acquired during the wet season of the year of 2009 in 11 separate flights between August 15 and September 10, using an Opetch ALTM 3010 sensor, and were collected by the Blom Corporation and Northrop–Grumman, as a part of an NSF project [26]. The average point density over the 50 ha plot is 10.8 point m^−2^.

For SdM the LiDAR data were collected in April 2012 by the GEOID Ltda. (Belo Horizonte, MG) as part of the Sustainable Landscapes Brazil joint project of the Brazilian Corporation of Agricultural Research (EMBRAPA) and the United States Forest Service (USFS). The study site was overflown with an Optech ALTM 3100 laser scanner instrument and an average point density of 20 m^−2^. More detailed information of the LiDAR data acquisition and flight parameters can be found in Leitold et al. [13].

#### Field measurements

For the SNM we collected 65 circular 0.09 ha (radius = 16.9 m) field plots using a stratified random sampling scheme using a Landsat-based pre-fire vegetation map provided by the U.S. Forest Service as reference. The strata were defined by vegetation type (softwood, hardwood, and mixed forests) and diameter classes (12.7–25.2; 25.2–50.6; 50.6–76 and >76 cm). For each tree with diameter at breast height (DBH) greater than 10 cm, the species was recorded and the DBH was tallied. The center of each plot was positioned using a differential GPS, with a horizontal accuracy after post-processing better than 0.5 m.

AGB was estimated using the National Biomass Estimator Library (NBEL), developed by the Forest Management Service Center (FMSC). Further information can be found in Garcia et al. [25].

The field measurements over BCI are confined to a 50 ha (1000 m × 50 m) plot managed by the CTFS. The field inventory data have been collected since 1982 [27] every 5 years. Here, we use the 2010 census data that includes all trees with a DBH greater than 10 cm, with measurements made higher on the bole for individuals with buttresses or trunk irregularities. The location of trees and its species was recorded allowing calculating the wood density (kg m^−3^) for every species. We included additional measurements on both DBH and individual tree height (TH) for 1604 individuals collected within the 50 ha plot during the dry season of 1993 and 1997 for allometric model development purposes [28]. It allows establishing a DBH-TH relationship to estimate TH in order to calculate the AGB for individual trees using Chave et al. [29] equations.

For the SdM, DBH of live individuals of tree and palm was measured in nine 1 ha permanent forest inventory plots established along an altitudinal transect in the area [23]. One plot is located in the lowland forest at an elevation of 100 m, four plots in the submontane forest between 180 and 370 m, and four plots in the montane forest at about 1000 m a.s.l [13]. A pantropical allometric model developed by Chave et al. [29] for tropical moist forests was applied to estimate the AGB for live trees (DBH > 4.8 cm, and for palms (DBH ≥ 4.8 cm), AGB was computed using the equation developed by Hughes [30].

### LiDAR processing

For each dataset the height of the returns was normalized using a digital elevation model (DEM) provided along with the datasets. After normalizing the datasets we derived 10 metrics that describe the vertical and horizontal distribution of vegetation and that are commonly used to estimate AGB from LiDAR data. These metrics included the mean height, percentiles of the height (25, 50, 75 and 90), the maximum height, the standard deviation, the coefficient of variation of the height distributions and the fractional cover. Finally we estimated the area under the canopy waveform as described in Garcia et al. [25]. These metrics were estimated by considering only canopy returns (h ≥ 2 m). For BCI, however, a threshold of 27 m was applied to compute FC after analyzing the relationship between crown area and biomass for that study area (Meyer, personal communication).

In addition, we derive the referred metrics using 1 m × 1 m canopy height models (CHM) for comparison purposes. The CHM is calculated by selecting the highest LiDAR point within each cell. However, due to the irregular spatial distribution of airborne LiDAR measurements the products might contain a significant number of empty cells, particularly those CHM calculated using low-density point clouds (e.g. 1 point m^−2^). Height information for empty cells is commonly derived using information from neighboring cells. Accordingly, we applied an interpolation method to generate the final CHM product. To do this, we first calculated a Delaunay triangulation using the LiDAR points that had been selected to compute the original CHM, i.e. the highest point within each cell. Then, we interpolated empty cells using the triangulation and a natural neighbor interpolation method.

In order to estimate the effect of point density on the metrics we thinned the original datasets to 10 points m^−2^ (SNM and SdM), 5 and 1 point m^−2^. The reduction of the point density was implemented by randomly removing points until the desired point density was achieved. Although this approach do not recreate the effects of changing survey configuration parameters (e.g. decrease of PRF or increase of flying height) on the return attributes and point cloud distribution that would occur under operational circumstances, it is a common approach to analyze the effect of point density on LiDAR metrics [11, 13, 31]. The same LiDAR metrics as for the original datasets were derived from the thinned datasets.

### Statistical analyses

To test the statistical significance of the differences for each of the metrics derived, we performed a two-sided Wilcoxon signed rank test. This non-parametric test was applied since the metrics tested showed a non-normal distribution (Kolmogorov–Smirnov test, p value <0.001). The two-sided Wilcoxon signed rank test examines the null hypothesis that the median difference between two samples is zero, against the alternative hypothesis that it is not. When evaluating the effect of point density, the metrics derived from the thinned datasets were evaluated against the metrics obtained from the original data. When evaluating the impact of plot size in the metrics, the reference data corresponded to the plot size used in the field measurements (0.09 ha for SNM and 1 ha for BCI and SdM). When the sample distribution for the difference between the corresponding metrics was not drastically different from normal, we also applied a two-sided one-sample t-test of the hull hypothesis that the mean value of the pair-wise difference between the metrics is zero. These t-tests yielded the same decisions as the corresponding Wilcoxon signed rank tests.

After analyzing the effect of point density and plot size on the metrics derived we fitted two exponential models to estimate AGB for each study site. The first exponential model used only the mean canopy height as explanatory variable. The second exponential model also included the fraction cover as it has been proved to improve results over different environments [2]. We selected mean canopy height and FC as explanatory variables because these are variables commonly used to model AGB. It should be borne in mind that our objectives were to evaluate the impact of data model selection—echo-based vs. CHM—and the point density on the metrics derived from LiDAR data. Therefore we did not attempt to find more complicated models or techniques (non-parametric approaches) that could help to improve our results.

## Abbreviations

AGB: aboveground biomass
CHM: canopy height model
DEM: digital elevation model
LiDAR: light detection and ranging
MCH: mean canopy height
REDD: reduced emissions from deforestation and forest degradation
RMSE: root mean square error
relRMSE: relative RMSE
